# Microglial APOE4: more is less and less is more

**DOI:** 10.1186/s13024-023-00693-6

**Published:** 2023-12-19

**Authors:** Ghazaleh Eskandari-Sedighi, Mathew Blurton-Jones

**Affiliations:** 1 Institute for Memory Impairments and Neurological Disorders, University of California Irvine, Irvine, CA 92697 USA; 2 Sue and Bill Gross Stem Cell Research Center, University of California Irvine, Irvine, CA 92697 USA; 3 Department of Neurobiology & Behavior, University of California Irvine, Irvine, CA 92697 USA

**Keywords:** Apolipoprotein E, Microglia, APOE4, APOE3, Alzheimer’s disease, Lgals3, TGFβ

## Abstract

Apolipoprotein E (APOE) is the single greatest genetic risk factor for late onset Alzheimer’s disease (AD). Yet, the cell-specific effects of APOE on microglia function have remained unclear. Fortunately, two comprehensive new studies published in the latest issue of *Nature Immunolog*y have employed complementary gain-of-function and loss-of-function approaches to provide critical new insight into the impact of microglial APOE on AD pathogenesis.

For over three decades, researchers have known that polymorphisms in Apolipoprotein E (APOE) represent the single greatest genetic risk factor for late-onset Alzheimer’s Disease (AD) [3]. Since then, the APOE4 risk allele has been shown to influence an array of diseased-associated processes including amyloid aggregation and clearance, tau-induced neurodegeneration, glucose metabolism, synaptic degeneration, and cerebral amyloid angiopathy (reviewed in detail in [9, 4], and [1]. Yet, the precise impact of APOE4 on microglial function has remained unclear. As a member of the apolipoprotein family of lipid binding proteins, human APOE is encoded by three differing alleles: ε2, ε3 and ε4. In comparison to the most common allele, ε3, a single copy of ε4 is associated with a threefold increased risk of developing AD, whereas two copies of ε4 leads to a remarkable 15-fold increase in disease risk [3]. In contrast, the ε2 allele confers a protective ~ sevenfold decreased risk of AD. The apoE protein has important roles in lipid homeostasis including the shuttling of cholesterol and lipids between cells. Each isoform also exhibits distinct binding affinities for different classes of lipoproteins. While astrocytes are the predominant producer of APOE within the healthy brain [10], microglia can significantly upregulate APOE expression in response to inflammatory insults, including AD pathology [5, 6, 8]. Understanding cell-specific roles of apoE isoforms within the brain will therefore provide invaluable new mechanistic insight that could likely inform the development of more effective therapeutics.

Fortunately, two highly complementary and comprehensive studies from the labs of Guojun Bu [7] and Oleg Butovsky [11], have now greatly advanced our understanding of the effects of APOE polymorphisms on microglial function. Led by co-first authors Liu and Wang, Yin and Rosenzweig, and published in *Nature Immunology*, each study applied opposing, yet highly complementary approaches to deconvolve the role of microglial APOE in AD pathogenesis. Using a Cx3cr1^creERT2/+^ mouse line to drive inducible expression of human APOE3 or APOE4 on a murine Apoe-knockout background, Liu et al. created a brain environment in which apoE3 or apoE4 could be specifically and exclusively expressed by microglia and CNS-associated macrophages (CAMs), also referred to as border-associated macrophages (BAMs) [7]. In contrast, Yin et al. crossed Cx3cr1^creERT2/+^ mice with mice expressing floxed versions of human APOE3 or APOE4, providing a paradigm to specifically delete APOE3 or APOE4 only within microglia and BAMs, while preserving human APOE expression in other cell types [11]. Both studies then crossed these inducible models with APP/PS1 mice to examine the impact of microglial-specific APOE4 or APOE3 expression [7] or deletion [11] on the microglial response to AD pathology. Despite their differing models and experimental designs, both groups reach the same ultimate conclusion: expression of human apoE4 restricts microglia in a more quiescent state, impairing their ability to mount a protective response to AD-associated pathology and increasing neurodegeneration (Fig. 1).

**Fig. 1:**
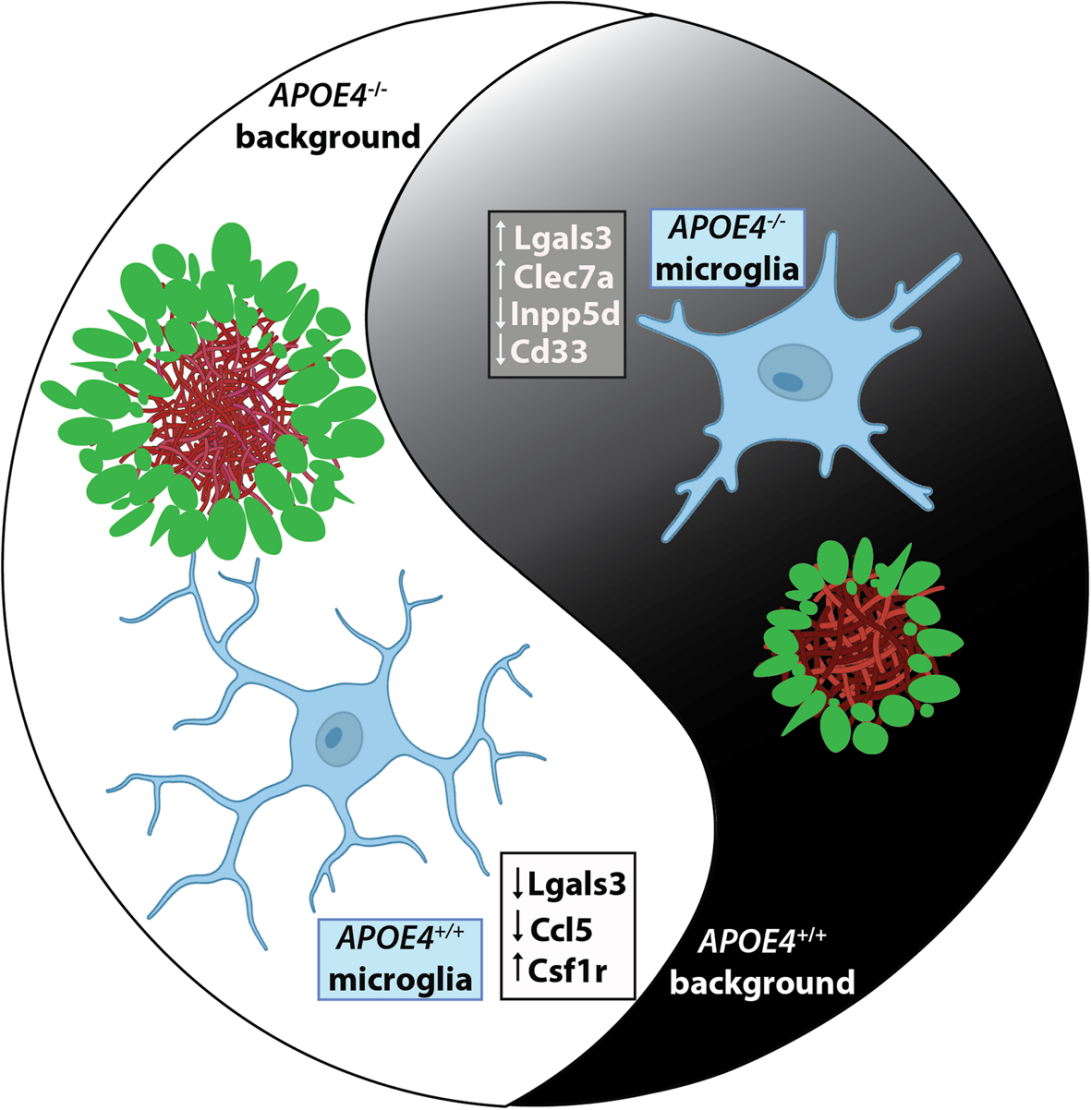
A schematic representation of the complementary gain-of-function and loss-of-function approaches used to examine the impact of human APOE on microglial function in AD. APOE4^+/+^ restricts microglia in a non-responsive state (left part of the circle), resulting in exacerbated amyloid pathology (red) and dystrophic neurites (green). In contrast, deletion of APOE4 in microglia rescues these deficits (right part of the circle)

Having validated the specificity of their model system, Liu et al. began by inducing microglial/CAM-specific expression of either apoE3 or apoE4 in 6-month-old APP/PS1 mice [7]. Three months later, they found that microglial apoE3 expression led to an increased number of plaque-associated microglia while concurrently reducing insoluble amyloid levels, dystrophic neurites, and microglial lipid droplets. Using single cell sequencing they further demonstrated that apoE3 induced a more reactive transcriptomic signature, promoting expression of neurodegenerative microglial (MGnD) transcripts. In contrast, microglial-specific expression of apoE4 produced opposing effects, worsening each of these pathological endpoints, impairing lipid metabolism, and promoting a stress-related microglial transcriptomic signature. Further analysis revealed apoE4-mediated induction of eIF2 signaling, oxidative phosphorylation and mitochondrial dysfunction in APP/PS1 mice, as well as downregulation of genes associated with the complement pathways, and lysosomal degradation.

Next, Liu and colleagues thoroughly assessed the cell-autonomous and cell-non-autonomous effects of microglial apoE on astrogliosis. Accordingly, they observed that in the absence of endogenous mouse Apoe, microglial expression of apoE3 reduced Aβ-associated cortical astrogliosis whereas apoE4 promoted astrocytic activation. While GFAP and Aβ levels exhibited a strong positive correlation in mice expressing microglial apoE3, no correlation was observed in apoE4 expressing mice, suggesting that microglial apoE4 can influence astrogliosis in an Aβ-independent manner. To further understand the potential impacts of Apoe expression in other cells they combined their inducible system with 5xFAD mice that express endogenous murine Apoe. In this model, the number of GFAP-positive astrocytes was again increased by microglial expression of apoE4, but no changes in GFAP were observed in 5xFAD mice expressing microglial apoE3 on a murine apoE expressing background. Taken together, the authors concluded that these differences are likely due to the cell-autonomous and non-cell-autonomous effects of apoE as well as the development of diffused versus compact amyloid plaques. Importantly, microglial apoE4 also influenced non-amyloid-dependent functions, reducing microglial response to a localized laser ablation, impairing cognition, and disrupting long term potentiation in comparison to apoE3 expressing microglia.

Taking an opposite approach, Yin and colleagues began their study by investigating the effects of global humanized apoE3 and apoE4 expression, finding that microglia isolated from apoE4 knockin mice exhibited increased expression of homeostatic genes in comparison to apoE3 mice [11]. When challenged by intracerebral injection of apoptotic neurons, apoE3 microglia mounted a robust phagocytic response, upregulating MGnD, interferon-responsive, and antigen presentation-associated transcripts, while apoE4 microglia failed to induce these disease-responsive genes. Furthermore, microglial-targeted deletion of apoE4 rescued the response to apoptotic neurons, elevating expression of MGnD genes while suppressing homeostatic transcripts.

To examine the effects of microglial-specific apoE deletion in the context of AD, Yin et al. also employed the APP/PS1 model as well as the P301S model of tauopathy [11]. Consistent with their apoptotic neuron results, microglia isolated from 9-month-old P301S:APOE3-KI mice exhibited an induction of several MGnD genes in response to tau pathology and a corresponding downregulation of homeostatic transcripts including Tgfb1 and Smad3. In contrast, P301S:APOE4-KI microglia exhibited a blunted MGnD response and increased TGFβ-signaling. Importantly, deletion of microglial APOE4 reversed many of these transcriptional deficiencies, reduced tau hyperphosphorylation and prevented cortical neuronal loss. Turning to the APP/PS1 model, the group found that APP/PS1:APOE3-KI mice readily adopt an MGnD signature, expressing high levels of Lgals3 and Clec7a, whereas apoE4-expressing microglia again exhibited an impaired MGnD response mediated via upregulation of microglial homeostatic checkpoint genes, including ITGB8 and Inpp5d. Microglial-specific deletion of APOE4 in turn restored the MGnD signature, increased plaque associated microglia and plaque compaction, and reduced both Aβ pathology and dystrophic neurites. They also reported an apoE4-mediated induction of ITGB8-transforming growth factor-β (TGFβ) signaling within microglia that drove upregulation of homoeostatic checkpoint genes, reducing the MGnD phenotype.

Given the role of astrocyte derived ITGB8 in TGFβ activation and Butovsky’s prior work showing the importance of TGFβ in microglial homeostasis [2], Yin and colleagues next examined the effects of manipulating ITGB8 expression. Using both knockout approaches and intracerebral injection of ITGB8 neutralizing antibody, they found that reduction of ITGB8 signaling led to enhanced Ab phagocytosis, elevated microglial expression of antigen presentation and interferon genes, increased GFAP and Clec7a, and decreased plaque load. Taken together, they concluded that the MGnD state is regulated by the reciprocal induction of APOE signaling and suppression of TGFβ and introduce the microglial APOE4–ITGB8–TGFβ pathway as a negative regulator of the neuroprotective microglial responses to AD pathology.

A mutual discovery of both studies is the convergence of microglial apoE-driven effects on Lgals3, the gene encoding galectin-3, a lectin with immunoregulatory functions. This encouraged both groups to examine the impact of microglial apoE on astrocytes. Using GFAP immunostaining, Liu et al. observed that microglial expression of apoE3 reduced Aβ-associated astrogliosis, whereas apoE4 promoted Aβ-independent astrocytic activation. However, Yin et al. reported decreased astrocytic activation in the presence of microglial apoE4. These differences could be due to distinct approaches each group used to quantify astrocyte activation as well as the differing apoE background of their mouse models. In Liu et al. the mice had no expression of apoE within astrocytes and the amyloid deposits were diffuse in nature. In contrast, the model used by Yin et al. included astrocytic expression of human apoE and exhibited compact amyloid plaques. Differences in plaque compaction can in turn effect the astrocytic response. Regardless of this minor difference, both studies support a critical role for microglial apoE in modulating microglia-astrocyte crosstalk that converges on microglial-derived Lgals3 signaling.

Lastly and importantly, both groups further validated their observations in postmortem human AD brain samples; Liu et al. investigated the plaque-associated microglia and observed a significant reduction in Lgals3-positive responsive microglia in *APOE4* carriers compared to *APOE3*. Yin et al. used publicly available RNAseq datasets and reported lower levels of MGnD-associated genes as well as increased TGFβ signaling in *APOE4* carriers, further supporting their hypothesis that ITGB8–TGFβ signaling could influence the development of AD by inhibiting MGnD induction. Taken together, these back-to-back studies used complementary gain-of-function and loss-of-function approaches to provide critical new insight into the role of microglial apoE in AD, ultimately finding that when it comes to microglial APOE4, more is less and less is more.

## Data Availability

Not applicable.

## Abbreviations

APOE: Apolipoprotein E
AD: Alzheimer’s disease
CAMs: CNS-associated macrophages
BAMs: Border-associated macrophages
KI: Knockin
TGFβ: ITGB8-transforming growth factor-β
MGnD: Microglial neurodegenerative phenotype

## References

[1] Bu G (2022). APOE targeting strategy in Alzheimer’s disease: lessons learned from protective variants. Mol Neurodegeneration.

[2] Butovsky O, Jedrychowski M, Moore C (2014). "Identification of a unique TGF-β–dependent molecular and functional signature in microglia. Nat Neuroscience.

[3] Corder EH (1993). Gene dose of apolipoprotein E type 4 allele and the risk of Alzheimer's disease in late onset families. Science.

[4] Fernández-Calle R, Konings SC, Frontiñán-Rubio J (2022). APOE in the bullseye of neurodegenerative diseases: impact of the APOE genotype in Alzheimer’s disease pathology and brain diseases. Mol Neurodegeneration.

[5] Keren-Shaul H (2017). A unique microglia type associated with restricting development of Alzheimer’s disease. Cell.

[6] Krasemann S (2017). The TREM2-APOE pathway drives the transcriptional phenotype of dysfunctional microglia in neurodegenerative diseases. Immunity.

[7] Liu CC, Wang N, Chen Y, et al. Cell-autonomous effects of APOE4 in restricting microglial response in brain homeostasis and Alzheimer’s disease. Nat Immunol. 2023;24:1854–66. 10.1038/s41590-023-01640-9 PMC11980647 37857825

[8] Mathys H (2019). Single-cell transcriptomic analysis of Alzheimer’s disease. Nature.

[9] Raulin AC, Doss SV, Trottier ZA (2022). ApoE in Alzheimer’s disease: pathophysiology and therapeutic strategies. Mol Neurodegeneration.

[10] Xu Q (2006). Profile and regulation of apolipoprotein E (ApoE) expression in the CNS in mice with targeting of green fluorescent protein gene to the ApoE locus. J Neurosci.

[11] Yin Z, Rosenzweig N, Kleemann KL, et al. APOE4 impairs the microglial response in Alzheimer’s disease by inducing TGFβ-mediated checkpoints. Nat Immunol. 2023;24:1839–53. 10.1038/s41590-023-01627-6 PMC10863749 37749326

